# Applications of Artificial Intelligence in Corneal Nerve Images in Ophthalmology

**DOI:** 10.3390/diagnostics16040602

**Published:** 2026-02-18

**Authors:** Raul Hernan Barcelo-Canton, Mingyi Yu, Chang Liu, Aya Takahashi, Isabelle Xin Yu Lee, Yu-Chi Liu

**Affiliations:** 1 Institute of Ophthalmology and Visual Sciences, School of Medicine and Health Sciences, Tecnologico de Monterrey, San Pedro Garza Garcia 66278, Mexico; 2 Regenerative Therapy Group, Singapore Eye Research Institute, Singapore 169856, Singapore; 3 Cornea and Refractive Surgery Group, Singapore Eye Research Institute, Singapore 169856, Singapore; 4 Department of Cornea and External Eye Disease, Singapore National Eye Centre, Singapore 169856, Singapore; 5 Ophthalmology and Visual Sciences Academic Clinical Program, Duke-NUS Medical School, Singapore 169857, Singapore

**Keywords:** artificial intelligence, in vivo confocal microscopy, corneal nerves, neuropathy, deep learning

## Abstract

Corneal nerves (CNs) are essential to maintain corneal epithelial integrity and ocular surface homeostasis. In vivo confocal microscopy (IVCM) enables the acquisition of high-resolution visualization of CNs, allowing visualization on a microscopic level. Traditionally, CN images must be analyzed by manual examination, which is time consuming and labor intensive. Artificial intelligence (AI) has facilitated reliable analysis of CN parameters, allowing for automatic and semiautomatic analysis of CNs. These include the identification, segmentation, and quantitative analysis of various CN parameters. This review summarizes the applications of AI-driven, automatic, and semiautomatic models in the CN analysis of IVCM images while also focusing on their diagnostic relevance in dry eye disease (DED) and neuropathic corneal pain (NCP). Recent advancements in AI have transformed IVCM image analysis by improving reproducibility and reducing operator dependency and time. The AI-based algorithm has been demonstrated to have good performance and sensitivity to identify and quantify the CN metrics. AI has also been utilized to improve the diagnostic accuracy of DED with IVCM scans, involving multiple portions of the CNs, such as the inferior whorl region. When employed with IVCM images of patients with NCP, AI-assisted identification of microneuromas and changes in CN metrics has provided an improvement in diagnostic accuracy. Despite promising advances and outcomes, the widespread implementation of these AI models in CN image analysis requires large-scale validation. Future integration of multimodal AI algorithms remains a promising endeavor to enhance diagnostic accuracy and disease stratification.

## 1. Background

The cornea is the most densely innervated surface tissue in the human body [1,2]. Corneal sensory innervation originates primarily from the ophthalmic division of the trigeminal nerve. In addition to these sensory fibers, the cornea also receives autonomic innervation, comprising sympathetic fibers arising from the superior cervical ganglion and parasympathetic fibers derived from the ciliary ganglion [2]. CNs serve multiple essential functions, including mediating sensory input to pain, touch, and temperature; preserving epithelial homeostasis; and initiating reflexes such as tear production and blinking [1]. CNs have been demonstrated to engage in bidirectional interactions with both the nervous and immune systems by modulating local responses through the secretion of neuropeptides and closely associating with resident dendritic cells [3]. CN impairment and dysfunction result in corneal hypoesthesia and anesthesia, hallmark features of neurotrophic keratopathy [4,5]. Further damage to the sensory innervation diminishes the lacrimal reflex and impairs corneal epithelial repair mechanisms, ultimately leading to epithelial breakdown, ulceration, and potential perforation [4]. These highlight the critical role of CNs in maintaining epithelial integrity and ocular surface homeostasis.

IVCM is a noninvasive technique that permits real-time viewing of the corneal structure at the cellular level. It employs the principle of confocal imaging, emitting a point of light into the tissue, and then, the reflected light is captured via a pinhole aperture, therefore reducing the field of view, resulting in reduced image contrast and improved quality [6]. The inherent capabilities of IVCM allow for high-resolution visualization of CNs with up to 600 magnification [6]. The advances of analytic software further facilitate detailed analysis of the CN morphology and enable quantitative assessment of parameters such as corneal nerve fiber density (CNFD), corneal nerve fiber length (CNFL), tortuosity, and branching [7]. Direct visualization of the CN plexus is invaluable not only for monitoring CN alterations in corneal diseases but also for allowing for the exploration of the relationship between CN status and systemic neurodegeneration by analyzing small nerve fiber changes on corneas [6,8,9].

The application of AI in corneal images allows for automation and reproducibility of CN measurements and enhances the diagnostic precision of corneal pathology. AI has been employed to identify referable-level of pterygium [10], monitor the progression of corneal dystrophy, and enable early detection of ocular surface neoplasia [11,12] or Fuch’s endothelial dystrophy [13]. AI technology has accelerated workflows and reduced the time in image processing and analysis, mainly by models such as U-net, generative adversarial networks (GANs), and Res-Net, all using convolutional neural networks (CNNs) [14]. In this review, we focus on the application of AI in IVCM CN images in the context of image analysis, diagnosis, and differentiation of CN-related ocular surface diseases.

## 2. Methodology

A comprehensive literature search was conducted using the following databases: PubMed, Scopus, Web of Science, and Google Scholar. Boolean operators were applied to combine the following keywords: “in-vivo confocal microscopy”, “IVCM”, “Artificial Intelligence”, “AI”, “Deep Learning”, “Machine Learning”, “Corneal Nerves”, “Corneal neuropathy”, and “Corneal Nerve Pathology”. Medical Subject Headings (MeSH) terms were not used. Original research articles and review papers published in English that reported the application of AI for CN analysis in IVCM images were considered eligible. Studies specifically focused on the quantitative or qualitative assessment of CN morphology or applications of DED and NCP were included.

Publications dated between January 2010 and May 2025 were screened. The initial search identified 62 unique articles across all databases. Of those, 23 were excluded after title and abstract screening because they did not meet the scope of this review. Full-text evaluation and reference cross-checking yielded an additional six relevant studies. In total, 45 articles addressing AI applications in IVCM-based analysis and related neuropathic or ocular surface pathologies were included in this review.

## 3. Discussion

The application of artificial intelligence in the analysis of corneal nerve metrics.

While IVCM offers significant advantages for investigating corneal pathology, it is often time consuming to analyze just one image, with a single operator requiring 2–7 min [15]. As patient scans typically include multiple images across different corneal regions at different depths, comprehensive analysis of CNs can become labor intensive in routine clinical and research settings [16,17]. The development of automated or semiautomated AI tools for the correct analysis of CNs has allowed for a better understanding of IVCM scans. Automated segmentation is completely driven by algorithms and performs its tasks without operator dependency. Semiautomated analysis requires a certain degree of user guidance or manual corrections of the analysis. These can include selecting regions of interest, adjusting image segmentations or outputs, or adjusting thresholds. AI pipelines typically preprocess certain information about images before being analyzed by the AI. This can include noise reduction; contrast enhancements; removal of artifacts; scaling pixels; and rotating, flipping, or cropping images, while these tend to filter unwanted conditions, which can confuse the AI algorithm. Some of the earliest studies suggest the use of artificial models to simply identify the presence or absence of nerves [16]. One such tool, Liner 1.1, was developed by a research group in order to correctly identify nerves independently of the imaging device, local illumination, and media transparency [16]. It encompasses an automatic image recognition capable of detecting corneal nerves. While it builds on the framework of validation for future AI, it lacks optimization and performance metrics, which limit the tool for quantitative analysis. It was tested in two different types of IVCM imaging devices, which enhance reproducibility. The authors highlighted that employing automated analysis enables consistent nerve detection across various scenarios, including normal exposure, underexposure, overexposure, through scattered media, and in and outside the optical range of the device [17,18].

CN analysis usually begins with segmentation and then extracting the centerline of CN fibers, leading to tracing, which allows for quantitative data to be extracted from IVCM images [19]. Thus, these two steps are important in the analysis of CN morphological features [19]. Proposed models of automatic segmentation have been developed. Lin et al. proposed an automatic segmentation tool designed to deliver accurate results despite challenges such as inaccurate annotations, non-uniform illumination, and contrast variability [20]. Their model successfully segmented CN images while simultaneously enhancing image quality, facilitating consistent and reliable nerve analysis across variable imaging conditions. Their tool performed contrastive learning in segmentation pipelines and was validated against a baseline segmentation network. Some weaknesses of the study presented include the lack of transparent datasets and limited validation. Optimization of this system may include cross-validation to strengthen its validity. Another automatic model, developed by Wei et al., correctly segments images of CNs in a Chinese population [21], achieving an area under the curve (AUC) of 0.96, with a sensitivity of 96% and a specificity of 75%, with a segmenting speed at around 32 images per second. It analyzed IVCM images of 104 patients and was trained against labeled images from senior ophthalmologists. The AI application resulted in moderate specificity and lacked external cohort validation. Regarding tracing, an automatic recognition software was validated by Scarpa et al. in recognizing nerve fiber trajectory in IVCM images and compared against manual tracing [17]. The recognition software adequately performed CN tracing in 80.4% of healthy subjects and 83.8% of patients with corneal neuropathy [17]. It was tested in 90 images after performing independent validation of 80 normal images. It includes limitations like disease characterization, older methodology and limited performance metrics by modern standards. Another proposed deep learning (DL) module to trace CNs across images and mosaics, developed by Guimarães et al., achieved a sensitivity of 0.89 and correctly traced CNs in around 93% of images despite nerve tortuosity [18]. This AI method was tested in 246 human IVCM images from healthy subjects but lacked a deep learning architecture, which could limit its use on diverse pathologies. Additionally, external and multicenter validation would further aid in the use of this tool.

**Figure 1:**
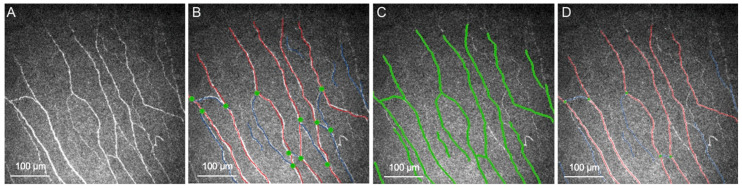
Illustrations of the CN analysis tools. Images presented at 600×. (**A**) Raw IVCM image of the subbasal CN plexus. (**B**) CN image marked manually by CCMetrics. (**C**) CN detection and (**D**) automated annotation by ACCMetrics. CN fibers are marked by red lines, nerve branches are marked by blue lines, and branch points are marked by green dots.

Correctly identifying, segmenting, and tracing nerves give way to analyzing quantitative measurements of CNs. One of the earliest applications of AI in IVCM images of CNs was to help in the segmentation of CNs and derive different parameters such as CNFL, CNFD, tortuosity, width, and corneal nerve branch density (CNBD) [22,23]. Some of the most widely used software for automatic detection, quantification, and analysis include CCMetrics and ACCMetrics (Weill-Cornell Medicine, Qatar & University of Manchester, Manchester, UK). CCMetrics is a semiautomated CN quantification software capable of measuring three main CN parameters, CNFD, CNBD, and CNFL, while also being capable of analyzing nerve tortuosity [15]. It has been validated on patients with diabetic neuropathy in multiple studies since its inception and has proved to be reliable in assessing adequate measurements of the previously mentioned parameters [24,25,26]. ACCMetrics is a fully automatic software for analyzing IVCM images, and in addition to the CCMetrics parameters, it integrates nerve fiber total branch density, nerve fiber area, nerve fiber width, and nerve fiber fractal dimension [7,15,27] (Figure 1). Both tools are widely used for research and diverse studies with multiple CN pathologies [21,22]. They have been validated in diverse populations and clinical scenarios, including patients with diabetes, refractive surgery, and DED. Results have proven that they are comparable to manual analysis of CNs.

Tortuosity of the CNs is a marker of nerve regeneration and neuroinflammation. It can result from diverse etiologies, such as diabetic corneal neuropathy, DED, NCP, and post-refractive surgery [28,29,30,31]. The presence of increased CN tortuosity usually translates into a state of active inflammation or neuropathic activity [31]. Estimating this finding is challenging as it reflects a combination of nerve curviness and waviness along their trajectory, compounded by the inherent variability in normal and pathological nerve morphology [32]. This complexity, along with branching, intersections, and anatomical variations, makes consistent and objective assessment difficult in IVCM images. Zhao et al. proposed a fully automated method for CN tortuosity analysis, which includes image enhancement, exponential curvature estimation, and tortuosity level classification [33]. They compared the automated pipeline of CN images to a dataset of 403 manually graded images to support their AI. The model can work around speckle noise, illumination inhomogeneities, and low contrast, culminating in better results comparable to expert human analysis, yet it lacks learned AI architecture like DL networks. A DL model was utilized by Mou et al. to quantify nerve tortuosity in 85.6% of IVCM images, which uses a double-staging method [34] where two models were used to segment the CNs first and then to quantify the tortuosity. They validated their DL, called DeepGrading, in around 1500 IVCM images and released their dataset to enhance reproducibility and benchmarking. This DL’s strengths include robust performance, results, and validation between healthy and diabetic groups, while certain limitations include reporting patient demographics and generalizability when analyzing images rather than fine segments.

The use of trained automated models can assist in accurately distinguishing nerves from other cellular structures, improving segmentation reliability [35]. Several automatic and AI models have been compared against manual analysis and segmentations. In a study performed in macaque monkeys, IVCM nerve images were automatically segmented by an AI model and compared with expert examiners [36]. The inter-class correlation coefficients between the model and the four examiners averaged 0.84, demonstrating a strong agreement with manual segmentation. Its major limitation remains the translation to human data and CN analysis and the limited sample size. This tool was also validated with a 5-fold cross-validation. As a preclinical model, this work represents a significant advancement in automated nerve segmentation. Another model utilized for CN segmentation was developed by Setu et al. [37] that was able to achieve a sensitivity of 86.1% and a specificity of 90.1% in automatic CN segmentation analysis. Inter-class correlation between manual and automatic segmentation was 0.85 for CN fiber numbers, 0.87 for CNFL, 0.95 for corneal branching, and 0.88 for nerve tortuosity, suggesting good agreement with the ground truth. They also provided a 10-fold cross-validation, ensemble voting, and tailored architecture while developing the AI model while reducing analysis time. It has been validated with multicenter data. Potential limitations include limited devices for testing, the use of cross-validation for the initial development, and disease variability. Another open-sourced automatic segmentation software, SNP-Net (Duke University, Durham, NC, USA), is publicly available. It achieved a DICE coefficient, the statistical measurement to evaluate the overlap between two sets of data, of 0.81 for CN segmentation when compared with manual validation [38]. It allows for multi-class segmentation, which can be applied to the cornea vortex or other locations of the cornea. The first stage uses GAN trained on 470 images to address artifacts, and the second uses a subbasal nerve plexus segmentation network trained in a 21-fold validation of 207 manually labeled images. It is available as an open-source software that facilitates future reproducibility and research. Nonetheless, it relies on cross-validation rather than external validation, which could infer a limitation on the AI. ACCMetrics and CCMetrics have also been compared with manual ground truths in patients following refractive surgery. Researchers demonstrated the accuracy of both automatic software when compared to manual annotations by expert analysis [39]. CNFD and CNFL showed the two best agreements, while CNBD demonstrated the poorest. Tang et al. proposed another model, called MLFGNet, for CN segmentation in IVCM images [40]. They evaluated the software in three independent datasets of images and achieved DICE coefficients of 89.3%, 89.4%, and 88.3% with each respective dataset, showing that the MLFGNet model accurately segments nerve images. Strengths include the validation on three different image datasets with the integration of progressive guidance. However, the study lacks a detailed disclosure of the underlying image datasets, like the number of images or clinical conditions, and specifics of training and validation. Regarding automatic examination of CN tortuosity, another AI model was further applied in patients with a history of refractive surgery [41]. The Spearman’s correlation between automatic and manual evaluation based on the Olivera-Soto and Efron scale of tortuosity [42] was 0.49, demonstrating a moderate correlation with possible room for improvement. This AI automated image analysis provided great clinical discrimination, allowing differentiation of patients with ocular pain from controls and OSDI grading. Limitations include a small, homogenous population and dataset validation and reliance on handcrafted processing steps, which could be sensitive to image quality. Scarpa and collaborators developed an automatic software to correctly evaluate corneal tortuosity [43]. Images were manually graded into low, mid, and high tortuosity and compared to the automatic measurement by the software. The results of corneal measurements and automatic grading were compared, with a concordance coefficient of 0.96. This model did not require machine learning and was validated in a dataset of 30 images from both normal and pathological subjects. Limitations of this study include the small dataset, lack of training on individual samples, and absence of noise handling.

Yildiz et al. proposed another DL algorithm based on GANs for CN segmentation [44]. GANs are considered deep learning models of two neural networks: generators and discriminators. Generators create synthetic images, and discriminators evaluate authenticity for segmentations. This automation was compared to a U-Net model for CN segmentation for CNFL measurements. U-Nets are convolutional neural networks (CNN) that are specifically designed for image segmentation. They analyze pixels to identify structures of interest from the image background. They encode and decode image features to precisely locate and segment fine structures. Both models demonstrated similar results, with a strong correlation coefficient of 0.85. Examiners added noise simulation to IVCM images (salt and pepper, speckle, and signal-to-noise) to simulate daily challenges. While the U-net model had lower accuracy when paired with noise, the GAN model did not decrease accuracy. Limitations include the inherent GAN training challenges and the lack of external device validation. This work provides strong evidence that GANs can enhance automated IVCM CN segmentations. Other automatic tools have been developed that can help in further understanding CNs in IVCM images. A thinning algorithm to aid in the extraction of the centerline of CN fibers, called neighborhood-statistics thinning, was developed [19]. Neighborhood-statistics thinning outperformed traditional methods by utilizing a tidal-cutting algorithm capable of narrowing the pixels, a tracing algorithm, and a CN fitting step to further improve the accuracy by aligning the extracted centerline with the CN fibers. It does not require training on raw IVCM labeled images but rather uses DL networks to develop. It demonstrated a precision rate of 0.82, able to extract the centerline with precision. The study’s reliance on this masking rather than the IVCM training could provide a certain degree of limitation to the device’s reproducibility.

Furthermore, CN image montages are useful since they provide a wider field of images, allowing a more detailed understanding of CN changes. A fully automated montaging tool to create wide-field montages of IVCM scans was developed [45]. This automated tool processes individual IVCM images and creates a wide-field image of the nerve subplexus. The software achieved a specificity of 100%, meaning no falsely matched images. It also reduced the manual workload by 57%, managing to create images in an average time between 1.5 and 3 h when compared to 10–20 h for manual montage reconstructions. While wide-field visualization permits faster and reliable observation of IVCM images, this study lacks testing on pathologic corneas, which could impair the montage of images. A novel tool called “NerveStitcher” aids in automatically creating overlapping images of the CNs following their trajectory through various IVCM images [46]. The software creates a stitch from 25 IVCM images, expanding the area analyzed by around six times. Similar to the previous tool, validation across pathological IVCM scans remains a valuable future endeavor to improve usage.

Table 1 summarizes the literature on the application of AI in the identification, segmentation, and quantification of CNs in IVCM images. In summary, AI-driven algorithms for automated segmentation and quantification on CN images provide a reliable assessment of CN parameters in a range of corneal diseases. They can achieve good results even in the context of background noise, anatomical variability, and image quality. These models have demonstrated a good correlation when compared with manual ground truth.

### 3.1. The Application of AI in Corneal Nerve Images in Dry Eye Diseases

**Figure 2:**
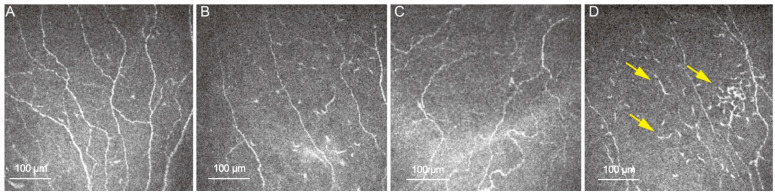
Representative IVCM images at 600× from a healthy subject and a patient with DED. (**A**) Normal CN morphology in a healthy individual. (**B**) CN abnormalities in DED, showing a decreased CN density and fiber length. (**C**) Increased CN tortuosity in DED. (**D**) Increased immune cells in DED (arrows).

DED is defined as a multifactorial ocular surface disease characterized by a loss of homeostasis of the tear film, instability, hyperosmolarity, neurosensory abnormalities, ocular surface inflammation, and damage, which are accompanied by ocular symptoms [47]. In IVCM scans, patients with DED presented with decreased CNFD, increased nerve tortuosity, increased number of bead-like formations, hyper-reflective activated keratocytes, and immune cells [47,48] (Figure 2). The presence of inflammatory cells in IVCM strongly correlates with the inflammation changes in DED. These changes usually include microstructural alterations like activation of intrastromal keratocytes, immune cell migration and infiltration, and an increase in basal epithelial hyperreflectivity [49,50]. IVCM has been employed to further investigate risk factors for DED and has been utilized to evaluate treatment response [51].

Researchers have utilized ACCMetrics to compare IVCM images of 39 patients with DED against 30 healthy controls [52]. They compared ACCMetrics values between DED and the control group using non-parametric tests, and the diagnostic performance was validated using ROC curves and AUC calculations. DED patients had significantly lower CNFD, CNBD, and CNFL compared with controls. CNFW demonstrated the highest diagnostic power, with an AUC of 0.828, for distinguishing DED from healthy controls. Sensitivity was 97.4%, and specificity was 46.7% for discriminating DED. While this study has many strengths, like automated quantification, large scale cohorts and clinical relevance, several limitations still arise, including its low specificity and the utilization of only ACCMetrics as the AI software. Replicating the results using other automated software could provide further insight on the differences between DED and healthy patients in IVCM scans.

Researchers further investigated the change patterns in corneal subbasal nerve morphology analyzed by AI and corneal intrinsic aberrations in DED [53]. They analyzed images from 155 patients with DED and 20 healthy controls. The AI technique utilized DL models to derive quantitative data from the IVCM scans. The authors found that corneal aberrations were significantly increased, and CNFD was notably decreased in the DED population. The CN average density was negatively correlated with the anterior, posterior, and total corneal aberrations, especially the higher-order aberrations. This study had strengths that include a large clinical cohort and the use of AI to understand corneal morphology. It also lacked in-depth details of the AI methodology, and it does not provide segmentation accuracy, such as DICE coefficients.

Additionally, Yu and colleagues built a novel semiautomatic software for the evaluation of the inferior whorl region of the cornea of patients with DED [54]. They validated the application with training and annotations. They evaluated 50 patients with DED and 30 control patients for IVCM parameters. The model achieved a DICE coefficient of 77.6% and an AUC of 0.98 in distinguishing patients with DED from those without it. The semiautomatic process also identified a decrease in CNFL, branching points, and fractal dimension in patients with DED [50,51]. Several limitations include the single-center dataset, which limits the demographics and image variability and generalizability, a focused field of views and not segmenting immune cells separately.

IVCM permits a noninvasive insight into cellular-level changes in patients with DED. Quantitative software has further enhanced diagnostic accuracy and is able to identify the changes in nerve quantity and morphology. These advancements support the use of AI in IVCM as a valuable assistant for the diagnosis, monitoring, and evaluation of DED patients.

### 3.2. The Application of AI in Corneal Nerve Images in Neuropathic Corneal Pain

**Figure 3:**
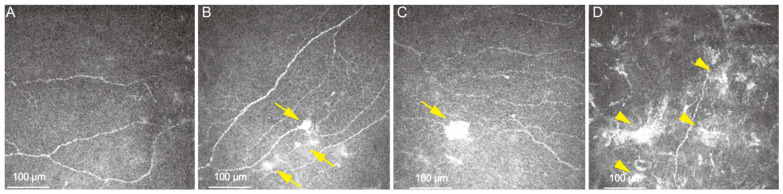
Representative IVCM images at 600× from a patient with NCP. (**A**) Decreased CN density and CN fiber length in NCP. (**B**,**C**) Arrows show different numbers and types of microneuromas in NCP patient manifested as irregularly shaped enlargements of terminal nerve endings with poorly defined margins and variable hyper-reflectivity. (**D**) Arrows show activated keratocytes, presenting as patchy areas of increased reflectivity in the corneal stroma.

NCP results from impaired CN function, causing aberrant nociception and hypersensibility of the CNs. This leads to disproportionate ocular pain, discomfort, and symptoms such as a burning sensation, foreign body sensation, and photophobia [55]. Diagnosis can be challenging, as no international consensus on the definition of NCP exists. A careful medical history, along with no significant clinical signs in the ocular surface, often points out the first main signs of NCP [55,56]. On IVCM, patients with NCP present with a reduced CNFD, activated keratocytes, increased corneal epithelial size, and the presence of microneuromas [55,57] (Figure 3). While not pathognomonic for NCP, the presence of microneuromas has been identified as a potential diagnostic feature of NCP [55,57,58]. Studies have demonstrated that microneuromas are present in nearly all eyes with NCP [55,57]. Although they can also occur in eyes without NCP, microneuromas in NCP typically exhibit greater length, perimeter, and total area compared to those in healthy eyes and DED [55,59]. IVCM’s capability of identifying microneuromas has enhanced the possibility of distinguishing between DED and NCP. Patients with microneuromas and ocular pain assessment survey scores showed high diagnostic accuracy when distinguishing both entities, with a reported AUC of 0.916 [59].

AI models have been developed to support the diagnosis of NCP. The first, proposed by Koseoglu et al., was designed to detect the presence or absence of microneuromas in IVCM images [60]. The model was trained internally using 82,359 images from 51 patients, validated using cross-validation procedures, and subsequently evaluated on an independent external dataset of 20,809 images from 100 patients. Performance metrics included the area under the precision–recall curve (AuPRC), area under the receiver operating characteristic curve (AuROC), and F1-score. The model achieved an AuROC of 0.966 on internal validation and was closely matched by an AuROC of 0.907 on the external validation cohort, underscoring its potential clinical utility in detecting microneuromas in patients with suspected NCP. The detection of microneuromas showed high predictive value for NCP against patients with DED. However, a key limitation is that both the internal and external datasets consisted exclusively of patients with either NCP or DED, thereby limiting generalizability to cases involving other corneal or systemic pathologies.

A recent study investigated whether AI-based quantification of IVCM images of the inferior whorl region of the subbasal nerve plexus could effectively distinguish patients with NCP from DED [61]. The study analyzed IVCM scans from 160 eyes between DED patients, NCP patients and healthy controls using a DL algorithm that quantitatively assessed six morphological parameters: nerve area density, average nerve thickness, nerve tortuosity, junction point density, neuroma density, and immune cell density. Their AI model previously developed utilized CNN and GANs to automatically segment and extract quantitative data. The authors reported a significantly reduced nerve area density in both DED and NCP groups compared to healthy individuals, while junction point density was notably lower in the NCP group relative to both controls and the DED group [61]. DED also showed higher immune cell density, which was not observed as prominently in NCP. These findings highlight a potential use of AI in distinguishing between the two diseases by analyzing CNs in the inferior whorl. This study provides many strengths, including the comparison of DED, NCP, and controls, which allows for comparisons between groups and the detection of immune cells and neuromas. Limitations include the lack of retraining the previous model, the absence of direct segmentation metrics in this cohort, and the cross-sectional study design.

Although not specifically designed for the diagnosis of NCP, a multimodal AI framework was developed to evaluate ocular surface pain by integrating IVCM findings with clinical and symptomatic data [62]. A total of 151 patients were evaluated by retrospective chart analysis: 120 patients presenting with primary ocular surface pain or discomfort and 31 control patients. Patients were assessed using the Ocular Surface Disease Index (OSDI), Schirmer’s test, systemic disease screening, and detailed IVCM analysis, including the quantification of CN fiber morphology, microneuroma presence, and dendritic cell density. These variables were input into a random forest classifier, which stratified subjects into four groups based on the concordance between symptoms and clinical signs: Group 1 (symptoms > signs), Group 2 (symptoms = signs), Group 3 (signs > symptoms), and Group 4 (healthy controls). The AI analysis revealed that ocular surface pain was most strongly associated with the presence of microneuromas within the subbasal nerve plexus, followed by the density of mature and immature dendritic cells. The model demonstrated high classification accuracy in Groups 1 (81.1%), 2 (86.3%), and 4 (79.7%), while accuracy decreased in Group 3 (69.1%), likely due to the presence of neurotrophic corneas or reduced corneal sensitivity in this subgroup [62]. The multimodal approach in this study and different groups of symptomatic and asymptomatic patients are strengths, while the model limitations are only using a random forest, lack of external validation, and moderate performance. This study highlights the potential of AI-driven, multimodal models to reclassify ocular surface pain phenotypes by incorporating subjective symptoms and objective biomarkers, advancing diagnostic precision beyond conventional clinical evaluation alone.

Furthermore, the use of AI permits the possibility of distinguishing between DED and NCP by identifying subtle changes and inflammatory patterns, especially in the subbasal nerve plexus, which cannot be discernible through conventional clinical examination or symptom assessment alone. Although both conditions may exhibit reduced CN structural parameters, NCP is more characteristically associated with the presence of microneuromas, a highly sensitive indicator of aberrant nerve regeneration, while DED more commonly demonstrates increased immune cell infiltration, reflecting its predominant inflammatory pathophysiology.

In summary, IVCM provides a good insight into NCP, especially in the detection of microneuromas. By incorporating AI models, it achieves high accuracy in detecting microneuromas and analyzing CN parameters to aid in diagnosing NCP while also giving an insight into CN structural changes and symptom severity.

### 3.3. Limitations and Future Directions

While AI in IVCM has allowed for a faster, reliable, and reproducible form of analyzing CNs, limitations still exist in its use. One such limitation is the generalizability of many DL models. Training of those models usually uses the datasets from a single population, which may provide different reproducibility in other ethnicities or populations. Most automatization models are tested on a single disease type or condition; hence, understanding how they would be utilized or behave in a different setting is warranted. While most DL models must be trained beforehand, the information fed into them can alter the performance in other groups of patients, with the possibility of the models being trained with other groups beforehand. The overfitting of data can also cause an overestimation when compared to larger populations. The use of multicenter and large-scale validation is imperative to the clinical translation of these programs in wider populations and health care centers. Each study represented its own type of validation, with some of them being cross-validation, which induces high variance across small datasets with no external cohort validation. Cross-validation’s performance in only the original dataset does not guarantee external validation or reproducibility in different populations or diseases. It could introduce data leakage or optimistic bias, leading to overestimation of the performance.

While several automatic models utilize different approaches to improve low image quality analysis or enhancement of the images, many programs are still prone to image quality for good results. While IVCM is still a user-dependent modality, a good operator is needed to be able to obtain good image resolution to be correctly analyzed by many automated programs. Additionally, while there is a normative database on CN parameters based on age [63], further research is required to define sex-adjusted, race-adjusted, and demographic-specific normative variation in CN metrics.

Currently, most of these software and AI applications are used after image acquisition. Future work would focus on the integration of these algorithms into clinical settings to aid clinicians in real-time diagnostic workflow and decision-making. The cost-effectiveness of incorporating AI into clinical implementation must also be taken into consideration.

AI algorithms incorporating multimodal data are an emerging approach that allows for better diagnostic accuracy and enhances decision-making [64]. Multimodal AI analysis allows for a better understanding of the condition and aids in customizing treatment and planning [62]. Further validation of new AI models to be utilized in other CN pathologies also remains a promising future direction.

## 4. Conclusions

AI provides an innovative tool for the analysis of CNs on IVCM images. The current work shows a potential to characterize CN morphology, refine image analysis, aid in disease classification, and improve the diagnostic precision of CN pathologies. Current applications have relevance for conditions such as DED and NCP. Future advancements will require multicenter validation, standardization of the reference metrics, and development of more expandable and reliable AI tools.

## Figures and Tables

**Table 1:** Applications of AI in identification, segmentation, and quantification of CNs in IVCM.

Study	Main Findings
Identification	
Avetisov [16]	Correctly identifies nerves independently of the imaging device, local illumination and media transparency
Segmentation/Tracing	
Lin [20]	Also allows for enhancing image quality
Wei [21]	Tested on IVCM images of Chinese populations to segment CN. It achieved an AUC 0.96 for segmenting CNs in the subbasal plexus.
Scarpa [17]	Correctly performed CN imaging tracing in healthy subjects and patients with corneal pain
Guimarães [18]	Correctly traced nerves in high tortuosity IVCM images
Quantification	
Chen [22]	Quantification of CNFL, CNFD, CNBD, and tortuosity. Tested on healthy controls and patients with diabetes. Achieved overall sensitivity of 91.7%.
CCMetrics [15,24,25,26]	Semiautomated CN quantification tool for CNFL, CNFD, CNBD and nerve tortuosity
ACCMetrics [7,15,24,25,26,27]	Fully automated CN quantification tool for same values as CCMetrics and total branch density, nerve fiber area, fiber width and fractal dimension
Zhao [33]	Developed to quantify CN tortuosity and tested in Chinese and Italian IVCM datasets of both healthy subjects and patients with diabetes and DED
Mou [34]	Tested in a 1500 image dataset to quantify CN tortuosity. It was able to correctly quantify CN tortuosity in around 85.64% of all images.
Comparisons of quantification models	
Oakley [36]	Comparison between examinators and AI automatic model of IVCM images of macaque monkeys. Inter-class correlation was 0.84, showcasing a strong agreement.
Setu [37]	Comparison of AI model with manual segmentation. Achieved a sensitivity of 86.1% and a specificity of 90.1% when compared to manual segmentation.
Zemborain [38]	Automatic segmentation software validated against several sets of IVCM images and compared against manual validation. DICE coefficient of 0.81 when comparing against manual validation.
Chin [39]	Application of CCMetrics and ACCMetrics compared to manual analysis of CN segmentation in refractive surgery patients; CNFD and CNFL showed the two best agreements, while CNBD demonstrated the poorest.
Tang [40]	Automatic model for CN segmentation validated in three sets of IVCM images. Achieved DICE coefficients of 89.3%, 89.4% and 88.3% with each dataset.
Fernandez [41]	Comparison of CN tortuosity in refractive surgery patients when compared to manual analysis. Showed a spearman correlation of 0.49 against manual grading, demonstrating room for improvement.
Scarpa [43]	Comparison of CN tortuosity against manual gradings. Results showed a concordance coefficient of 0.96 between both sets of groups after being previously graded into low, mid or high tortuosity.
Yildiz [44]	Comparison between a generative adversarial network and a U-Net model for CN segmentation. Both models demonstrated similar results, with a strong correlation coefficient of 0.85. Authors added challenges to images including noise to simulate daily challenges.
Chen [19]	Comparison of a neighborhood-statistics thinning model and compared to other AI models. The automatic model achieved a precision rate of 0.82 when trying to extract the centerline rather than thinning.
Image Generation	
Turuwhenua [45]	Creates a widefield image of a CN subplexus from IVCM images
Li [46]	NerveSticher: generates an IVCM image of 25 overlapping images to better follow CN trajectory

## Data Availability

No new data were created for this study.

## Abbreviations

AUC	Area under the curve
AuPRC	Area under the precision–recall curve
AuROC	Area under the receiver operating characteristic curve
AI	Artificial intelligence
CNN	Convolutional neural networks
CN	Corneal nerve
CNBD	Corneal nerve branch density
CNFD	Corneal nerve fiber density
CNFL	Corneal nerve fiber length
DL	Deep learning
DED	Dry eye disease
GAN	Generative adversarial networks
IVCM	In vivo confocal microscopy
MeSH	Medical Subject Headings
NCP	Neuropathic corneal pain
OSDI	Ocular Surface Disease Index
